# Supplementary material for: Assessment of burden and segregation profiles of CNVs in patients with epilepsy
Source: Ann Clin Transl Neurol. 2022 Jun 8;9(7):1050–8. doi: 10.1002/acn3.51598 (PMC9268881; doi:10.1002/acn3.51598)
Supplement: Supplementary file 1 — Figure S1 LRR's PCA before (left panel) and after (right panel) PC correction. Symbols represent the different batches and colors of the different plates (many plates were sent for genotyping within one batch). Figure S2. Pedigree of the mixed patient (DEE in a NAFE family) carrying the de novo deletion. Unaffected individuals are in black. [file ACN3-9-1050-s001.docx]

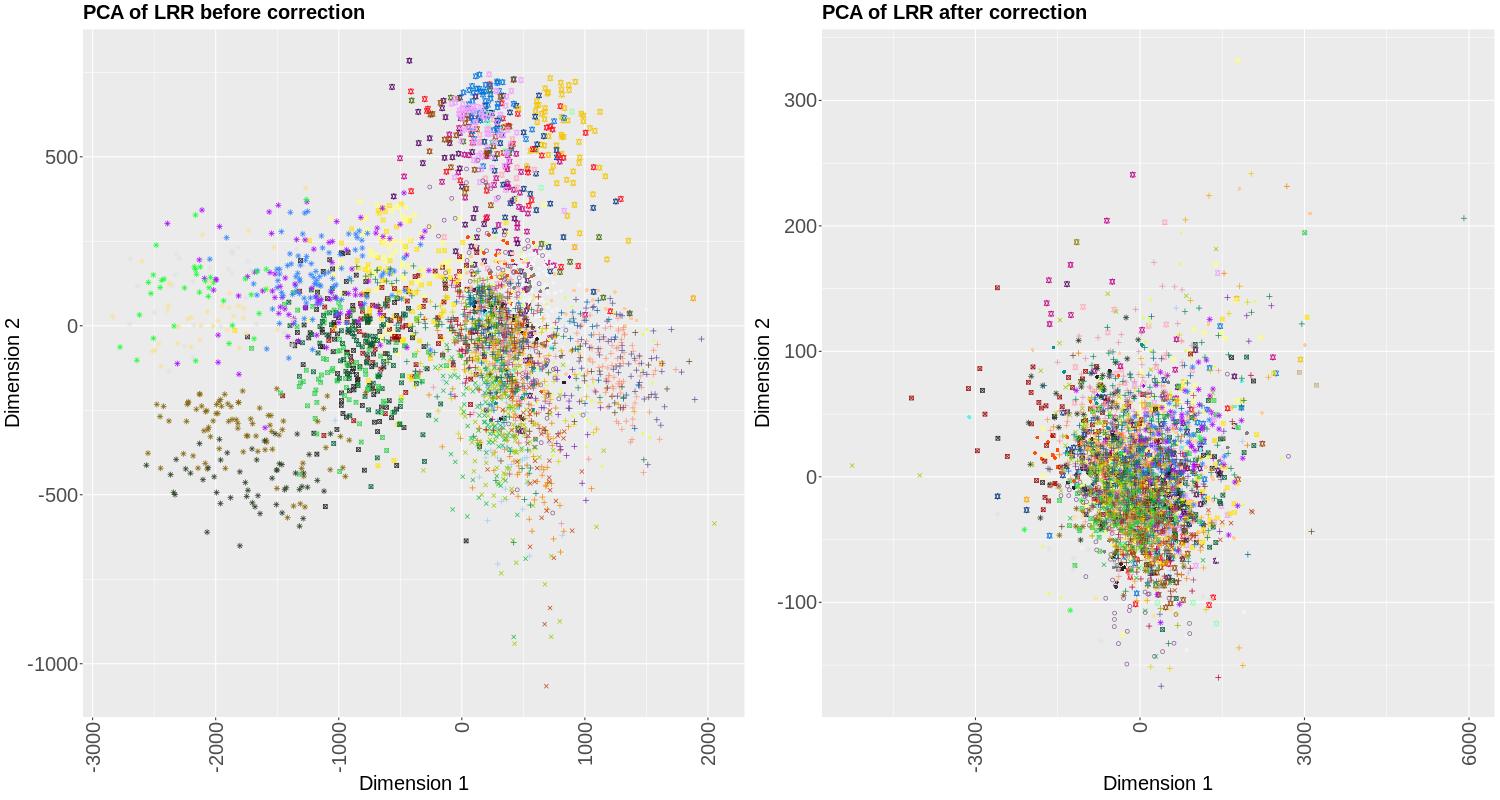


**Supplementary figure 1:** LRR’s PCA before (left panel) and after (right panel) PC correction. Symbols represent the different batches and colors the different plates (many plates were sent for genotyping within one batch).


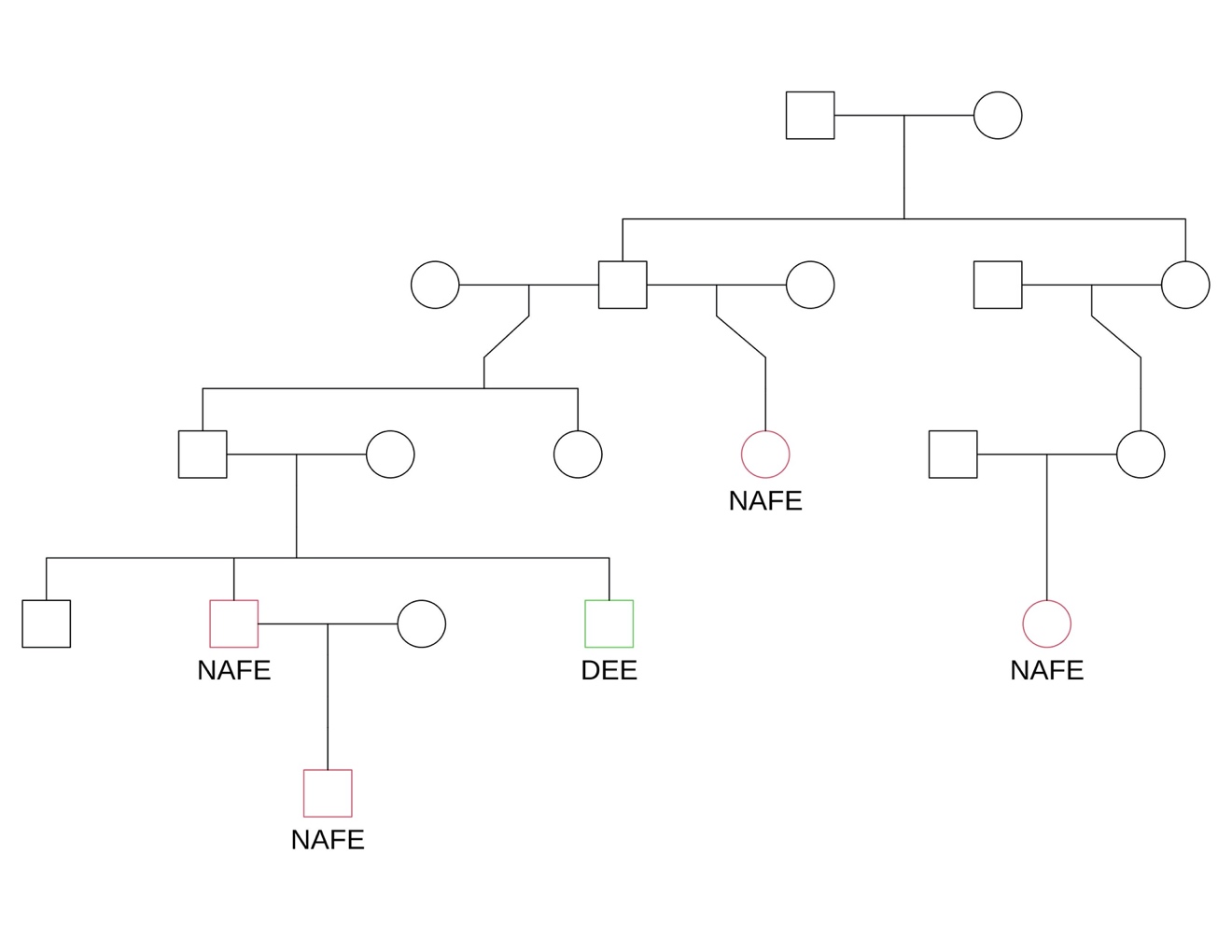


**Supplementary Figure 2:** Pedigree of the mixed patient (DEE in a NAFE family) carrying the *de novo* deletion. Unaffected individuals are in black.
