# Supplementary material for: Assessment of burden and segregation profiles of CNVs in patients with epilepsy
Source: Ann Clin Transl Neurol. 2022 Jun 8;9(7):1050–8. doi: 10.1002/acn3.51598 (PMC9268881; doi:10.1002/acn3.51598)
Supplement: Supplementary file 3 — Table S2 Recurrent deletions' description from Watson et al. [file ACN3-9-1050-s007.docx]

**Supplementary Table 2:** Recurrent deletions’ description from Watson *et al.* ^14^

| **Chromosomal location** | **Coordinates hg19** | **OMIM** | **Epilepsy/candidate gene** | **Selected reference** |
| --- | --- | --- | --- | --- |
| 1q21.1 | Chr1 :146.5-147.5 |  | *GJA8* | ^1–3^ |
| 2q21.1 | chr2:131.48-131.9 |  |  | ^4^ |
| 7q11.23 distal | chr7:75.07-76.25 | 613729 |  | ^5^ |
| 10q11.21–q11.23 | chr10:42.5-52.5 |  |  | ^6^ |
| 15q11.2 | chr15:21-25.5 | 615656 | *NIPA1,CYFIP1* | ^2,7^ |
| 15q11–q13 | chr15:23-28.5 |  |  | ^8^ |
| 15q13.3 | chr15:31.5-33.5 | 612001 | *CHRNA7* | ^9,10^ |
| 16p11.2 | chr16:28.5-34 | 611913 | *PRRT2* | ^11^ |
| 16p13.11 | chr16:14.9-16.6 |  | *NDE1* | ^7,12^ |
| 17q12 | chr17:32-37.5 | 614527 |  | ^13^ |
| 22q11.2 | chr22:18-25.5 | 611867 | *SNAP29* | ^3^ |

**References**

1. Stone JL, O’Donovan MC, Gurling H, et al. Rare chromosomal deletions and duplications increase risk of schizophrenia. Nature 2008;

2. Mefford HC, Muhle H, Ostertag P, et al. Genome-wide copy number variation in epilepsy: novel susceptibility loci in idiopathic generalized and focal epilepsies. PLoS genetics 2010;6(5):e1000962.

3. Lal D, Ruppert AK, Trucks H, et al. Burden Analysis of Rare Microdeletions Suggests a Strong Impact of Neurodevelopmental Genes in Genetic Generalised Epilepsies. PLoS Genetics 2015;11(5):e1005226.

4. Dharmadhikari A V., Kang SHL, Szafranski P, et al. Small rare recurrent deletions and reciprocal duplications in 2q21.1, including brain-specific ARHGEF4 and GPR148. Human Molecular Genetics 2012;

5. Ramocki MB, Bartnik M, Szafranski P, et al. Recurrent distal 7q11.23 deletion including HIP1 and YWHAG identified in patients with intellectual disabilities, epilepsy, and neurobehavioral problems. American Journal of Human Genetics 2010;

6. Stankiewicz P, Kulkarni S, Dharmadhikari A V., et al. Recurrent deletions and reciprocal duplications of 10q11.21q11.23 including CHAT and SLC18A3 are Likely Mediated by Complex Low-Copy Repeats. Human Mutation 2012;

7. De Kovel CGF, Trucks H, Helbig I, et al. Recurrent microdeletions at 15q11.2 and 16p13.11 predispose to idiopathic generalized epilepsies. Brain 2010;133(1):23–32.

8. Moreno-De-Luca D, Sanders SJ, Willsey AJ, et al. Using large clinical data sets to infer pathogenicity for rare copy number variants in autism cohorts. Molecular Psychiatry 2013;

9. Helbig I, Mefford HC, Sharp AJ, et al. 15q13.3 microdeletions increase risk of idiopathic generalized epilepsy. Nature Genetics 2009;41(2):160–162.

10. Dibbens LM, Mullen S, Helbig I, et al. Familial and sporadic 15q13.3 microdeletions in idiopathic generalized epilepsy: Precedent for disorders with complex inheritance. Human Molecular Genetics 2009;18(19):3626–3631.

11. Weiss LA, Shen Y, Korn JM, et al. Association between Microdeletion and Microduplication at 16p11.2 and Autism. New England Journal of Medicine 2008;

12. Heinzen EL, Radtke RA, Urban TJ, et al. Rare Deletions at 16p13.11 Predispose to a Diverse Spectrum of Sporadic Epilepsy Syndromes. American Journal of Human Genetics 2010;86(5):707–718.

13. Mefford HC, Clauin S, Sharp AJ, et al. Recurrent reciprocal genomic rearrangements of 17q12 are associated with renal disease, diabetes, and epilepsy. American Journal of Human Genetics 2007;

14. Watson CT, Marques-Bonet T, Sharp AJ, Mefford HC. The genetics of microdeletion and microduplication syndromes: An update. Annual Review of Genomics and Human Genetics 2014;15:215–244.
