# Supplementary material for: Assessment of burden and segregation profiles of CNVs in patients with epilepsy
Source: Ann Clin Transl Neurol. 2022 Jun 8;9(7):1050–8. doi: 10.1002/acn3.51598 (PMC9268881; doi:10.1002/acn3.51598)
Supplement: Supplementary file 4 — Table S3 Deletions in epilepsy genes. [file ACN3-9-1050-s003.pdf]

Supplementary Table 3: Deletions in epilepsy genes.

CNV	Length	Genes	Phenotype	Sex	Family*	Coding	In intolerant gene	In epilepsy gene	Hotspot
chr1:44174081-44446651	272571	ARTN,ATP6V0B,B4GALT2,DPH2,IPO13,ST3GAL3,U6	GGE	1	6231	1	0	1	0
chr14:67146763-67486808	340046	GPHN	GGE	2	6233	1	1	1	0
chr15:30936285-32514341	1578057	AK093758,ARHGAP11B,CHRNA7,DKFZp434L187,DQ572979,DQ582939,DQ588973,DQ595055,DQ596686,DQ600342,DQ786280,FAN1,HERC2P10,JB175342,KLF13,LOC100288637,LOC283710,MIR211,MTMR10,OTUD7A,TRPM1	fam_ctrl	2	6001	1	0	1	15q13.3
chr15:30936285-32514341	1578057	AK093758,ARHGAP11B,CHRNA7,DKFZp434L187,DQ572979,DQ582939,DQ588973,DQ595055,DQ596686,DQ600342,DQ786280,FAN1,HERC2P10,JB175342,KLF13,LOC100288637,LOC283710,MIR211,MTMR10,OTUD7A,TRPM1	fam_ctrl	2	6001	1	0	1	15q13.3
chr15:30936285-32514341	1578057	AK093758,ARHGAP11B,CHRNA7,DKFZp434L187,DQ572979,DQ582939,DQ588973,DQ595055,DQ596686,DQ600342,DQ786280,FAN1,HERC2P10,JB175342,KLF13,LOC100288637,LOC283710,MIR211,MTMR10,OTUD7A,TRPM1	GGE	1	6001	1	0	1	15q13.3
chr15:30936285-32514341	1578057	AK093758,ARHGAP11B,CHRNA7,DKFZp434L187,DQ572979,DQ582939,DQ588973,DQ595055,DQ596686,DQ600342,DQ786280,FAN1,HERC2P10,JB175342,KLF13,LOC100288637,LOC283710,MIR211,MTMR10,OTUD7A,TRPM1	GGE	2	6001	1	0	1	15q13.3
chr15:30936285-32514341	1578057	AK093758,ARHGAP11B,CHRNA7,DKFZp434L187,DQ572979,DQ582939,DQ588973,DQ595055,DQ596686,DQ600342,DQ786280,FAN1,HERC2P10,JB175342,KLF13,LOC100288637,LOC283710,MIR211,MTMR10,OTUD7A,TRPM1	GGE	2	6087	1	0	1	15q13.3
chr15:30936285-32514341	1578057	AK093758,ARHGAP11B,CHRNA7,DKFZp434L187,DQ572979,DQ582939,DQ588973,DQ595055,DQ596686,DQ600342,DQ786280,FAN1,HERC2P10,JB175342,KLF13,LOC100288637,LOC283710,MIR211,MTMR10,OTUD7A,TRPM1	GGE	1	6282	1	0	1	15q13.3
chr15:30936285-32514341	1578057	AK093758,ARHGAP11B,CHRNA7,DKFZp434L187,DQ572979,DQ582939,DQ588973,DQ595055,DQ596686,DQ600342,DQ786280,FAN1,HERC2P10,JB175342,KLF13,LOC100288637,LOC283710,MIR211,MTMR10,OTUD7A,TRPM1	GGE	1	6360	1	0	1	15q13.3
chr15:30936285-32514341	1578057	AK093758,ARHGAP11B,CHRNA7,DKFZp434L187,DQ572979,DQ582939,DQ588973,DQ595055,DQ596686,DQ600342,DQ786280,FAN1,HERC2P10,JB175342,KLF13,LOC100288637,LOC283710,MIR211,MTMR10,OTUD7A,TRPM1	GGE	2	6328	1	0	1	15q13.3
chr15:30936285-32514341	1578057	AK093758,ARHGAP11B,CHRNA7,DKFZp434L187,DQ572979,DQ582939,DQ588973,DQ595055,DQ596686,DQ600342,DQ786280,FAN1,HERC2P10,JB175342,KLF13,LOC100288637,LOC283710,MIR211,MTMR10,OTUD7A,TRPM1	GGE	1	6430	1	0	1	15q13.3
chr16:29652488-30192359	539872	AB209061,AK097453,AK097472,AK097527,ALDOA,ASPHD1,BC029255,BC041466,BOLA2,C16orf54,C16orf92,CDIPT,CDIPT-AS1,DOC2A,FAM57B,GDPD3,HIRIP3,INO80E,KCTD13,KIF22,MAPK3,MAZ,MVP,PAGR1,PPP4C,<br>PRRT2,QPRT,SEZ6L2,SPN,TAOK2,TBX6,TMEM219,YPEL3,ZG16	Mixed	2	6067	1	1	1	16p11.2
chr16:79095848-79121835	25988	WWOX	ctrl	2	NA	1	0	1	0
chr16:79095848-79121835	25988	WWOX	DEE_trio_parent	1	264	1	0	1	0
chr16:79130574-79171137	40564	WWOX	fam_ctrl	2	6029	1	0	1	0
chr16:79130574-79171137	40564	WWOX	GGE	2	6029	1	0	1	0
chr16:79130574-79171137	40564	WWOX	GGE	1	6029	1	0	1	0
chr2:50918967-51033295	114329	NRXN1	ctrl	2	NA	1	1	1	0
chr22:32129550-32240460	110911	DEPDC5,PRR14L	fam_ctrl	2	4007	1	1	1	0
chr22:32129550-32240460	110911	DEPDC5,PRR14L	NAFE	2	4007	1	1	1	0
chr22:32129550-32255802	126253	DEPDC5,PRR14L	fam_ctrl	1	4007	1	1	1	0
chr22:33760604-34150984	390381	LARGE,LARGE-AS1,MIR4764,SNORA50	fam_ctrl	1	4013	1	0	1	0
chr22:33760604-34157526	396923	LARGE,LARGE-AS1,MIR4764,SNORA50	NAFE	2	4013	1	0	1	0
chr22:33760604-34172801	412198	LARGE,LARGE-AS1,MIR4764,SNORA50	ctrl	2	NA	1	0	1	0
chr22:33760604-34172801	412198	LARGE,LARGE-AS1,MIR4764,SNORA50	fam_ctrl	2	4031	1	0	1	0
chr22:34096135-34194745	98611	LARGE,LARGE-AS1,SNORA50	ctrl	2	NA	1	0	1	0
chr22:34096135-34194745	98611	LARGE,LARGE-AS1,SNORA50	NAFE	1	4027	1	0	1	0
chr22:34096135-34196626	100492	LARGE,LARGE-AS1,SNORA50	ctrl	2	NA	1	0	1	0
chr7:147011388-147067719	56332	CNTNAP2,MIR548I4	ctrl	1	NA	1	0	1	0
chr8:17888770-17977230	88461	ASAH1	GGE	2	6315	1	0	1	0

Red = epilepsy genes from:

Berkovic SF, Scheffer IE, Petrou S, et al. A roadmap for precision medicine in the epilepsies. The Lancet Neurology 2015;14(12):1219–28.

Coppola A, Cellini E, Stamberger H, et al. Diagnostic implications of genetic copy number variation in epilepsy plus. Epilepsia 2019;60(4):689–706.

* Note that family numbers have been recoded for privacy concerns
