# Supplementary material for: Assessment of burden and segregation profiles of CNVs in patients with epilepsy
Source: Ann Clin Transl Neurol. 2022 Jun 8;9(7):1050–8. doi: 10.1002/acn3.51598 (PMC9268881; doi:10.1002/acn3.51598)
Supplement: Supplementary file 5 — Table S4 Deletions in epilepsy hotspots. [file ACN3-9-1050-s002.pdf]

Supplementary Table 4: Deletions in epilepsy hotspots.

CNV	Length	Genes	Phenotype	Sex	Family*	Coding	In intolerant ge	In epilepsy gen	Hotspot
chr15:241122	41909	NOT_FOUND	GGE	1	6124	0	0	0	15q11.2
chr15:243696	98818	AK124131,PWRN2	ctrl	1	NA	1	0	0	15q11.2
chr15:243696	98818	AK124131,PWRN2	ctrl	2	NA	1	0	0	15q11.2
chr15:243696	98818	AK124131,PWRN2	ctrl	2	NA	1	0	0	15q11.2
chr15:243696	98818	AK124131,PWRN2	ctrl	1	NA	1	0	0	15q11.2
chr15:243696	98818	AK124131,PWRN2	DEE	1	2102	1	0	0	15q11.2
chr15:243696	98818	AK124131,PWRN2	DEE_trio_pare	1	2102	1	0	0	15q11.2
chr15:243696	98818	AK124131,PWRN2	fam_ctrl	1	6395	1	0	0	15q11.2
chr15:243696	98818	AK124131,PWRN2	GGE	2	6212	1	0	0	15q11.2
chr15:243696	98818	AK124131,PWRN2	GGE	2	6344	1	0	0	15q11.2
chr15:243696	98818	AK124131,PWRN2	GGE	1	6433	1	0	0	15q11.2
chr15:243696	279962	AK124131,PWRN2	NAFE	2	4231	1	0	0	15q11.2
chr15:243766	91839	AK124131,PWRN2	ctrl	1	NA	1	0	0	15q11.2
chr15:259620	56982	ATP10A	ctrl	1	NA	1	0	0	15q11–q13
chr15:309362	1578057	AK093758,ARHGAP11B,CHRNA7,DKFZp434L187,DQ572979,DQ582939,DQ588973,DQ595055,DQ596686,DQ600342,DQ786280,FAN1,HERC2P10,JB175342,KLF13,LOC100288637,LOC283710,MIR211,MTMR10,OTUD7A,TRPM1	fam_ctrl	2	6001	1	0	1	15q13.3
chr15:309362	1578057	AK093758,ARHGAP11B,CHRNA7,DKFZp434L187,DQ572979,DQ582939,DQ588973,DQ595055,DQ596686,DQ600342,DQ786280,FAN1,HERC2P10,JB175342,KLF13,LOC100288637,LOC283710,MIR211,MTMR10,OTUD7A,TRPM1	fam_ctrl	2	6001	1	0	1	15q13.3
chr15:309362	1578057	AK093758,ARHGAP11B,CHRNA7,DKFZp434L187,DQ572979,DQ582939,DQ588973,DQ595055,DQ596686,DQ600342,DQ786280,FAN1,HERC2P10,JB175342,KLF13,LOC100288637,LOC283710,MIR211,MTMR10,OTUD7A,TRPM1	GGE	1	6001	1	0	1	15q13.3
chr15:309362	1578057	AK093758,ARHGAP11B,CHRNA7,DKFZp434L187,DQ572979,DQ582939,DQ588973,DQ595055,DQ596686,DQ600342,DQ786280,FAN1,HERC2P10,JB175342,KLF13,LOC100288637,LOC283710,MIR211,MTMR10,OTUD7A,TRPM1	GGE	2	6001	1	0	1	15q13.3
chr15:309362	1578057	AK093758,ARHGAP11B,CHRNA7,DKFZp434L187,DQ572979,DQ582939,DQ588973,DQ595055,DQ596686,DQ600342,DQ786280,FAN1,HERC2P10,JB175342,KLF13,LOC100288637,LOC283710,MIR211,MTMR10,OTUD7A,TRPM1	GGE	2	6087	1	0	1	15q13.3
chr15:309362	1578057	AK093758,ARHGAP11B,CHRNA7,DKFZp434L187,DQ572979,DQ582939,DQ588973,DQ595055,DQ596686,DQ600342,DQ786280,FAN1,HERC2P10,JB175342,KLF13,LOC100288637,LOC283710,MIR211,MTMR10,OTUD7A,TRPM1	GGE	1	6282	1	0	1	15q13.3
chr15:309362	1578057	AK093758,ARHGAP11B,CHRNA7,DKFZp434L187,DQ572979,DQ582939,DQ588973,DQ595055,DQ596686,DQ600342,DQ786280,FAN1,HERC2P10,JB175342,KLF13,LOC100288637,LOC283710,MIR211,MTMR10,OTUD7A,TRPM1	GGE	2	6328	1	0	1	15q13.3
chr15:309362	1578057	AK093758,ARHGAP11B,CHRNA7,DKFZp434L187,DQ572979,DQ582939,DQ588973,DQ595055,DQ596686,DQ600342,DQ786280,FAN1,HERC2P10,JB175342,KLF13,LOC100288637,LOC283710,MIR211,MTMR10,OTUD7A,TRPM1	GGE	1	6360	1	0	1	15q13.3
chr15:309362	1578057	AK093758,ARHGAP11B,CHRNA7,DKFZp434L187,DQ572979,DQ582939,DQ588973,DQ595055,DQ596686,DQ600342,DQ786280,FAN1,HERC2P10,JB175342,KLF13,LOC100288637,LOC283710,MIR211,MTMR10,OTUD7A,TRPM1	GGE	1	6430	1	0	1	15q13.3
chr15:329229	48988	NOT_FOUND	fam_ctrl	1	4013	0	0	0	15q13.3
chr16:150927	132606	FLJ00285,NPIP,NTAN1,PDXDC1,RRN3	GGE	2	6406	1	0	0	16p13.11
chr16:154930	798938	ABCC1,ABCC6,AX747846,C16orf45,FOPNL,KIAA0430,MIR484,MPV17L,MYH11,NDE1	GGE	2	6406	1	1	0	16p13.11
chr16:154930	2671653	ABCC1,ABCC6,AK310228,AX747757,AX747846,C16orf45,DQ586919,DQ596229,FOPNL,KIAA0430,MIR3179-2,MIR3180-3,MIR484,MPV17L,MYH11,Mir_548,NDE1,NOMO3,NPIP,PKD1P1,XYLT1	fam_ctrl	2	6108	1	1	0	16p13.11
chr16:154930	2671653	ABCC1,ABCC6,AK310228,AX747757,AX747846,C16orf45,DQ586919,DQ596229,FOPNL,KIAA0430,MIR3179-2,MIR3180-3,MIR484,MPV17L,MYH11,Mir_548,NDE1,NOMO3,NPIP,PKD1P1,XYLT1	fam_ctrl	1	6108	1	1	0	16p13.11
chr16:154930	2671653	ABCC1,ABCC6,AK310228,AX747757,AX747846,C16orf45,DQ586919,DQ596229,FOPNL,KIAA0430,MIR3179-2,MIR3180-3,MIR484,MPV17L,MYH11,Mir_548,NDE1,NOMO3,NPIP,PKD1P1,XYLT1	GGE	2	6108	1	1	0	16p13.11
chr16:154930	2671653	ABCC1,ABCC6,AK310228,AX747757,AX747846,C16orf45,DQ586919,DQ596229,FOPNL,KIAA0430,MIR3179-2,MIR3180-3,MIR484,MPV17L,MYH11,Mir_548,NDE1,NOMO3,NPIP,PKD1P1,XYLT1	GGE	2	6108	1	1	0	16p13.11
chr16:288256	216410	AK125489,ATP2A1,ATXN2L,CD19,LAT,LOC100289092,MIR4517,MIR4721,NFATC2IP,NPIPL1,RABEP2,SH2B1,SPNS1,TUFM	ctrl	2	NA	1	1	0	16p11.2
chr16:296524	539872	AB209061,AK097453,AK097472,AK097527,ALDOA,ASPHD1,BC029255,BC041466,BOLA2,C16orf54,C16orf92,CDIPT,CDIPT-AS1,DOC2A,FAM57B,GDPD3,HIRIP3,INO80E,KCTD13,KIF22,MAPK3,MAZ,MVP,PAGR1,PPP4C,PRRT2,<br>QPRT,SEZ6L2,SPN,TAOK2,TBX6,TMEM219,YPEL3,ZG16	Mixed	2	6067	1	1	1	16p11.2
chr17:336840	84165	SLFN11,SLFN12,SLFN13	ctrl	2	NA	1	0	0	17q12
chr17:336840	84165	SLFN11,SLFN12,SLFN13	ctrl	1	NA	1	0	0	17q12
chr17:336840	84165	SLFN11,SLFN12,SLFN13	ctrl	1	NA	1	0	0	17q12
chr17:336840	84165	SLFN11,SLFN12,SLFN13	ctrl	1	NA	1	0	0	17q12
chr17:336840	84165	SLFN11,SLFN12,SLFN13	ctrl	2	NA	1	0	0	17q12
chr17:336840	84165	SLFN11,SLFN12,SLFN13	ctrl	2	NA	1	0	0	17q12
chr17:336840	84165	SLFN11,SLFN12,SLFN13	GGE	2	6386	1	0	0	17q12
chr17:336840	84165	SLFN11,SLFN12,SLFN13	GGE	1	6396	1	0	0	17q12
chr17:336840	84165	SLFN11,SLFN12,SLFN13	GGE	2	6406	1	0	0	17q12
chr17:336840	84165	SLFN11,SLFN12,SLFN13	GGE	1	6417	1	0	0	17q12
chr17:336840	84165	SLFN11,SLFN12,SLFN13	NAFE	2	4300	1	0	0	17q12
chr22:181153	518055	BC064400,BCL2L13,BID,DQ570096,Em:AC008101.5,FLJ41941,MICAL3,MIR3198-1,MIR648,PEX26,TUBA8,USP18	GGE	2	6292	1	1	0	22q11.2
chr22:188894	2574241	7SK,AIFM3,AK129567,AK302545,ARVCF,BC033281,BC035867,BC127858,BCRP2,BX648073,C22orf29,C22orf39,CDC45,CLDN5,CLTCL1,COMT,CRKL,DGCR10,DGCR11,DGCR14,DGCR2,DGCR5,DGCR6,DGCR6L,DGCR8,DGCR9,<br>DQ571461,DQ574263,DQ585141,GNB1L,GSC2,HIRA,HV593096,HV593110,HV593127,HV593134,HV593135,HV593178,HV593183,JX456220,KIAA1653,KLHL22,LINC00895,LINC00896,LOC284865,LOC388849,LOC400891,<br>LOC729444,LZTR1,MED15,MIR1286,MIR1306,MIR185,MIR3618,MIR4761,MRPL40,Metazoa_SRP,Mir_649,P2RX6,P2RX6P,PI4KA,PI4KAP1,POM121L4P,PRODH,RANBP1,RIMBP3,RTN4R,SCARF2,SEPT5-GP1BB,SERPIND1,<br>SLC25A1,SLC7A4,SNAP29,TANGO2,TBX1,THAP7,THAP7-AS1,TMEM191A,TMEM191B,TRMT2A,TSSK2,TUBA3FP,TXNRD2,U84523,UFD1L,USP41,Y_RNA,ZDHHC8,ZNF74	Mixed	1	4016	1	1	0	22q11.2
chr22:223144	47891	AK131325,TOP3B	ctrl	2	NA	1	0	0	22q11.2
chr22:223144	64605	AK131325,TOP3B	ctrl	1	NA	1	0	0	22q11.2
chr22:223144	64605	AK131325,TOP3B	ctrl	1	NA	1	0	0	22q11.2
chr22:223144	64605	AK131325,TOP3B	ctrl	1	NA	1	0	0	22q11.2
chr22:223144	64605	AK131325,TOP3B	ctrl	1	NA	1	0	0	22q11.2
chr22:223144	64605	AK131325,TOP3B	GGE	2	6072	1	0	0	22q11.2
chr22:223144	64605	AK131325,TOP3B	Mixed	1	4244	1	0	0	22q11.2

Orange = Same individual

* Note that family numbers have been recoded for privacy concerns
