# Supplementary material for: Assessment of burden and segregation profiles of CNVs in patients with epilepsy
Source: Ann Clin Transl Neurol. 2022 Jun 8;9(7):1050–8. doi: 10.1002/acn3.51598 (PMC9268881; doi:10.1002/acn3.51598)
Supplement: Supplementary file 7 — Table S6 Unmasked missense variants. [file ACN3-9-1050-s005.pdf]

Supplementary Table 6: Unmasked missense variants.

Chromosome	Position	Aleternate allele	Phenotype	WGS/WES	CNET cohort frequency	gnomAD frequency	gnomAD clinical significance	UNEECON score
17	15234895	T	Mixed	WGS	0.25	0.21	NA	0.000081
18	74980809	A	NAFE	WES	1.00	0.97	NA	0.000109
18	72228124	G	NAFE	WES	1.00	0.99	NA	0.000111
13	24895559	G	GGE	WGS	0.74	0.76	NA	0.000442
16	16271357	C	GGE	WGS	1.00	0.99	benign	0.000522
3	158388780	C	GGE	WES	0.53	0.52	NA	0.000650
13	23898664	G	GGE	WGS	0.87	0.88	benign	0.000700
18	72114455	T	NAFE	WES	1.00	0.99	NA	0.000802
15	31362352	T	GGE	WES	0.81	0.81	benign	0.000931
15	31362352	T	GGE	WGS	0.80	0.81	benign	0.000931
15	31362352	T	GGE	WES	0.81	0.81	benign	0.000931
15	31362352	T	GGE	WES	0.81	0.81	benign	0.000931
15	31362352	T	GGE	WGS	0.80	0.81	benign	0.000931
15	31362352	T	GGE	WGS	0.80	0.81	benign	0.000931
18	74611127	G	NAFE	WES	0.96	0.95	NA	0.001479
3	158366900	A	GGE	WES	0.53	0.55	benign	0.001635
18	66504459	T	NAFE	WES	1.00	0.98	NA	0.001985
18	72103782	C	NAFE	WES	0.38	0.35	NA	0.002147
18	72201918	A	NAFE	WES	0.12	0.11	NA	0.004205
17	14139891	C	Mixed	WGS	1.00	0.98	NA	0.004650
18	72021717	C	NAFE	WES	1.00	1.00	NA	0.005075
13	24411772	C	GGE	WGS	0.99	0.99	NA	0.007212
18	76753768	G	NAFE	WES	0.82	0.84	NA	0.012774
18	66513615	G	NAFE	WES	0.38	0.38	NA	0.015114
15	31197564	A	GGE	WES	0.44	0.46	benign	0.017849
15	31197564	A	GGE	WES	0.44	0.46	benign	0.017849
15	31197564	A	GGE	WGS	0.43	0.46	benign	0.017849
18	67718688	G	NAFE	WES	0.95	0.91	benign	0.020459
18	72998899	A	NAFE	WES	0.03	0.03	benign	0.023956
18	70417396	T	NAFE	WES	1.00	1.00	NA	0.026219
18	77473127	T	NAFE	WES	0.18	0.14	benign	0.031721
3	157160196	G	GGE	WES	7.4e-04	1.6e-05	NA	0.047242
18	67871343	C	NAFE	WES	0.92	0.86	benign	0.064903
18	76753588	G	NAFE	WES	0.84	0.79	benign	0.107196
15	31776021	C	GGE	WGS	0.99	0.99	NA	0.107602
15	31776021	C	GGE	WGS	0.99	0.99	NA	0.107602
15	31776021	C	GGE	WGS	0.99	0.99	NA	0.107602
13	24798506	T	GGE	WGS	0.03	0.02	NA	0.119246
18	77246406	G	NAFE	WES	0.49	0.42	NA	0.142239
17	15142755	A	Mixed	WGS	0.09	0.08	benign	#N/A
17	15134175	G	Mixed	WGS	0.53	0.53	benign	#N/A
